# A W-Band Communication and Sensing Convergence System Enabled by Single OFDM Waveform

**DOI:** 10.3390/mi13020312

**Published:** 2022-02-17

**Authors:** Nazar Muhammad Idrees, Zijie Lu, Muhammad Saqlain, Hongqi Zhang, Shiwei Wang, Lu Zhang, Xianbin Yu

**Affiliations:** 1 College of Information Science and Electronic Engineering, Zhejiang University, Hangzhou 310027, China; nazar@zju.edu.cn (N.M.I.); 3150102348@zju.edu.cn (Z.L.); saqlain@zju.edu.cn (M.S.); zhanghongqi@zju.edu.cn (H.Z.); wsw@zju.edu.cn (S.W.); zhanglu1993@zju.edu.cn (L.Z.); 2 Zhejiang Laboratory, Hangzhou 311121, China

**Keywords:** communication and sensing, cyclic prefix interval (CPI), orthogonal frequency division multiplexing (OFDM), range extension

## Abstract

Convergence of communication and sensing is highly desirable for future wireless systems. This paper presents a converged millimeter-wave system using a single orthogonal frequency division multiplexing (OFDM) waveform and proposes a novel method, based on the zero-delay shift for the received echoes, to extend the sensing range beyond the cyclic prefix interval (CPI). Both simulation and proof-of-concept experiments evaluate the performance of the proposed system at 97 GHz. The experiment uses a W-band heterodyne structure to transmit/receive an OFDM waveform featuring 3.9 GHz bandwidth with quadrature amplitude modulation (16-QAM). The proposed approach successfully achieves a range resolution of 0.042 m and a speed resolution of 0.79 m/s with an extended range, which agree well with the simulation. Meanwhile, based on the same OFDM waveform, it also achieves a bit-error-rate (BER) $10^{-2}$, below the forward error-correction threshold. Our proposed system is expected to be a significant step forward for future wireless convergence applications.

## 1. Introduction

Starting from Marconi’s first transatlantic wireless transmission in 1899, wireless communication has been a crucial technology for developing today’s modern lifestyle. There is a wide range of potential applications in wireless communication and sensing areas, such as cellular devices [1], wireless local area networks (WLANs) [2], vehicular communications [3], security scanner, biological diagnosis, non-destructive detection, and radar imaging [4]. From a technological perspective, a converged system is expected to provide enormous benefits in terms of both spectrum efficiency and cost-effectiveness [5,6,7]. In the past, different waveforms have been used independently for implementing most wireless communication, and sensing functionalities [8,9]; consequently, the systems are bulky, energy consumable, and uneconomical. In this context, a unified waveform simultaneously serving communication and sensing has gained substantial interest [10]. So far, the orthogonal frequency division multiplexing (OFDM) technique is well known for its benefits for wireless communications, and has not only been adopted in numerous standards but is also considered as a strong candidate for future wireless communication systems (5G and beyond) [11,12]. More interestingly, the OFDM waveform has also been well documented for its effectiveness in radar applications [13,14,15]. Therefore, OFDM waveforms are promising for the convergence of communication and sensing [16,17,18,19,20].

The OFDM wireless communication technically requires inverse fast Fourier transform (IFFT) and fast Fourier transform (FFT) operations to transmit and receive data. The cyclic prefix interval (CPI), also known as a guard interval, makes OFDM transmission robust against multi-path radio channel. However, under the channel impulse response longer than the CPI, inter-symbol interference (ISI) degrades communication performance, and in mobility scenarios, inter-carrier interference (ICI) causes orthogonality loss among the subcarriers and ISI as a consequence. There are some approaches to equalize this issue in communication, for instance, basis-expansion-model-based channel transformation [21], iterative finite length-equalization technique [22], and adjusting the CPI length according to the channel length [23].

### 1.1. Related Works

The OFDM waveform for sensing can be processed either by the conventional correlation-based approach [24,25], or by OFDM symbol-based processing [26]. Correlation-based sensing is usually performed by cross-correlation in the delay and Doppler domains between the transmitted and received pulses, and different schemes have been proposed to improve sensing performance. For example, a good approximation of the transmitted signal is generated at the receiver for removing clutter in the correlation-based target detection [15]. Work in [25] proposes to use the information of data symbols for ambiguity suppression, and circular correlation for range extension up to an OFDM symbol duration. Different correlation-based OFDM radar receiver schemes have been compared in [27], in terms of complexity, signal-to-interference-plus-noise-ratio, and robustness against ground clutter.

Alternatively, similar to OFDM-based communication, OFDM-based sensing can also use IFFT/FFT operations to extract range and speed information. Based on this approach, a 77 GHz OFDM-based sensing system with a bandwidth of 200 MHz demonstrated a sensing resolution of 0.75 m with the maximum range of 150 m [28]. Another OFDM-based radar at 77 GHz used a stepped carrier approach to achieve a sensing resolution of 0.146 m with a bandwidth of 1.024 GHz, while the maximum range is 60 m [29]. Moreover, the authors implemented OFDM-based radar processing for automotive scenario by using a relatively longer interval of 128 ms to achieve speed resolution of 0.22 m/s, while the range resolution was 1.87 m for a bandwidth of 80 MHz at 5.2 GHz [30].

These two sensing processing approaches were employed in the development of OFDM-based radars, while from the viewpoint of converging OFDM-based communication and sensing, OFDM symbol-based sensing processing is more attractive, provided that a sensing receiver is synchronized with the transmitter and the transmitted data are readily available for sensing processing. Some interesting research has been done on OFDM-based convergence in the microwave band. By using OFDM waveforms which are designed for 3GPP-LTE and 5G-NR at 2.4 GHz with a bandwidth of 98.28 MHz, OFDM-based sensing supports a sensing resolution of 1.5 m and a maximum range of 350 m and performs an algorithm for self-interference cancellation in the full-duplex mode [31]. Authors in [32] provide measurement results for the indoor mapping using a 28 GHz carrier frequency for the 5G-NR with a bandwidth of 400 MHz and achieve a sensing resolution of 0.4 m. Another work in [33] shows results of mmWave demonstration testbed for joint sensing and communication; measurements were performed at 26 GHz with a bandwidth of 10 MHz to identify the angular location of different targets using beamforming technique. The work in [34] also presents a range resolution of 1.61 m and a maximum range of 206 m within 93 MHz bandwidth at the 24 GHz band. In addition, authors in [35] provide a parameter selection criterion for joint OFDM radar and communication systems by considering vehicular communication scenarios, such as CPI, subcarriers spacing, and coherence time of the channel.

### 1.2. Motivation and Contribution

Please note that enabling the sensing functionality of the OFDM waveform (which is designed for wireless communication) does not provide the flexibility of parameter adjustment according to the sensing requirements. Furthermore, the ISI cancellation/compensation techniques proposed for OFDM wireless communication are not differently applicable for OFDM-based sensing because the transformation or truncation-based equalization destructs the sensing information. Ideally, the delay of an echo for sensing should fall within the CPI, and the Doppler frequency normalized over OFDM waveform interval should be an integer. However, in a real scenario for sensing, a target is located randomly and moves with an arbitrary speed. Consequently, an OFDM waveform designed for communication shows limitation in obtaining high sensing resolution and a large detection range.

As we know, the detection range of a single target is determined by the detectable OFDM signal strength and an adjustment of delay offset. In the case of multiple echoes with delay beyond the CPI, the OFDM-based sensing is mainly limited by the ISI, free-space-path-loss (FSPL), and processing gain. Echoes outside the CPI cause ISI as previous OFDM symbols interfere with current OFDM symbol in the processing window, which increases the threshold for target detection. In addition, echoes with delay longer than the CPI will achieve less processing gain, which reduces linearly with the delay. This loss of processing gain along with the ISI makes it difficult for OFDM-based sensing to detect targets outside the CPI, particularly in the millimeter-wave region featuring large bandwidth and high FSPL. Therefore, the extension of sensing range beyond the conventional limit of CPI is one of the important issues in developing communication and sensing converged systems for applications such as indoor mapping, digital health monitoring, unmanned aerial vehicles, and residential security.

In this work, we propose and experimentally demonstrate a converged communication and sensing system operating at 97 GHz using the same 16-QAM (quardrature amplitude modulation) OFDM waveform. An approach based on zero-delay shift is proposed to extend the detectable range by compensating for the IFFT processing gain for echoes outside the CPI. In the proposed method, we extended the range of an OFDM-based sensing, while the simplicity of operations for range and speed estimation is achieved using IFFT/FFT operations. The proposed method uses delay-shifts in the received signal before processing a received OFDM symbol. Active subcarriers in the received OFDM symbol are divided by the active subcarriers in the current and previous transmitted OFDM symbols (employed number of transmitted OFDM symbols determine the rang extension), and IFFT operations are used after each delay-shift to generate matrices in the delay and delay-shift domains (delay domain is the result of IFFT operation). Delay-shift rows at delay zero are concatenated to extend the delay-shift domain. Concatenation of delay-shift rows for different received OFDM symbols provides a matrix in delay-shift and time domain, and FFT operations over time domain provide the speed estimation. An experiment with a heterodyne W-band transmitter/receiver is performed, and both sensing and communication performance are measured in terms of range/speed profile and bit-error-rate (BER). The proposed approach for range extension is verified for distances well beyond the CPI and provides a range resolution of 0.042 m, and speed resolution of 0.79 m/s using a single OFDM waveform, which is promising in driving OFDM-based converged systems for future applications.

The rest of this paper is organized as follows. Section 2 presents the model for the OFDM-based converged system to provide the details of extracting sensing information from the received OFDM waveform. Section 3 details the proposed method for range extension in an OFDM-based converged system. Section 4 provides simulation results, while Section 5 is dedicated to experimental measurement results and discussions. Section 6 provides the conclusion of this work.

## 2. Communication and Sensing Convergence Using OFDM Waveforms

Motivated by the OFDM-based sensing presented in [26,34], a reference system model for the convergence of communication and sensing is presented here. An OFDM waveform for communication purposes consists of several OFDM symbols, each with orthogonal subcarriers modulated by data symbols and cyclically extended by appending the last part of the signal at the beginning called cyclic prefix (CP). If Δf represents the subcarriers spacing, *N* the number of orthogonal subcarriers, *T* the OFDM symbol duration, $T_{\mathrm{cp}}$ as the CPI, $T_{s}=T_{\mathrm{cp}}+T$ the effective duration of the OFDM symbol, and *M* the number of OFDM symbols, then the analytical expression of the transmitted OFDM waveform is [34],

(1) $$s\left(t\right)=\sum_{\mu =0}^{M-1}\sum_{n=0}^{N-1}S\left(\mu N+n\right)e^{\left(j2\pi n\Delta f(t-\mu T_{s})\right)}e^{\left(-j2\pi n\Delta fT_{\mathrm{cp}}\right)}\mathrm{rect}\left(\frac{t-\mu T_{s}}{T_{s}}\right),$$

where S(μN+n) is the data symbol at *n*th subcarrier of μth OFDM symbol. The $\mathrm{rect}\left(t\right)$ in (1) is the rectangular pulse shape, such that rect(t)=1 for t∈[01] and 0 otherwise. The term $\exp\left(-j2\pi n\Delta fT_{\mathrm{cp}}\right)$ appears due to the cyclic extension of OFDM symbols by the CP.

In order to fulfill the orthogonality among subcarriers, over the interval *T*, the following condition must be held:

(2) $$\Delta f=\frac{1}{T},$$

and $T_{\mathrm{cp}}$ should accommodate the maximum expected delay caused by the radio channel. The baseband signal s(t) is up-converted by a carrier frequency $f_{c}$ to form $\tilde{s}\left(t\right)$ for transmission,

(3) $$\tilde{s}\left(t\right)=s\left(t\right)e^{j2\pi f_{c}t}.$$

The received signal $\tilde{a}\left(t\right)$ at the sensing receiver is the sum of echoes from different targets. Using point-target channel model for *L* number of targets,

(4) $$\tilde{a}\left(t\right)=\sum_{l=1}^{L}b_{l}\tilde{s}\left(t-\tau_{l}\right),$$

where $\tau_{l}$ and $b_{l}$ represent delay and attenuation related to the *l*th target, respectively. If *l*th target is located at a distance $R_{l}$ and moving with a speed of $v_{l}$, delay $\tau_{l}$ in the received echo can be expressed as

(5) $$\tau_{l}=\frac{2(R_{l}-v_{l}t)}{c_{0}},$$

and $b_{l}$ [34],

(6) $$b_{l}=\sqrt{\frac{c_{0}^{2}G_{\mathrm{Tx}}G_{\mathrm{Rx}}\sigma_{{\mathrm{RCS}}_{l}}}{{\left(4\pi \right)}^{3}R_{l}^{4}f_{c}^{2}}},$$

where in (6), $c_{0}$ is the speed of light in free space; $\sigma_{{\mathrm{RCS}}_{l}}$ is the radar cross-section of the *l*th target; and $G_{\mathrm{Tx}}$, $G_{\mathrm{Rx}}$ represent transmitting and receiving antenna gain, respectively.

For the communication link, the signal attenuation $b_{\mathrm{com}}$ is

(7) $$b_{\mathrm{com}}=\sqrt{\frac{c_{0}^{2}G_{\mathrm{Tx}}G_{\mathrm{Rx}}}{{\left(4\pi \right)}^{2}R_{\mathrm{com}}^{2}f_{c}^{2}}},$$

where $R_{\mathrm{com}}$ indicates the distance of the communication link.

For sensing processing, a single target is sufficient for mathematical derivations due to the linear operation in (1). The analytical expression for the received echo from a target, located at a distance *R*, moving with the speed of *v*, and attenuated by $\hat{b}$ (assuming constant attenuation factor for frequencies within the bandwidth) is obtained by using delay $\tau =\left(2R-2vt\right)/c_{0}$ in (1), i.e.,

(8) $$\begin{array}{cc} \tilde{a}\left(t\right) & =\sum_{\mu =0}^{M-1}\sum_{n=0}^{N-1}\hat{b}S\left(\mu N+n\right)e^{\left(j2\pi n\Delta f(t-\frac{\left(2R-2vt\right)}{c_{0}}-\mu T_{s}-T_{\mathrm{cp}})\right)}\\ & \;\cdot e^{\left(j2\pi f_{c}\left(t-\frac{\left(2R-2vt\right)}{c_{0}}\right)\right)}\mathrm{rect}\left(\frac{t-\left(\frac{2R-2vt}{c_{0}}\right)-\mu T_{s}}{T_{s}}\right)+\hat{z}\left(t\right), \end{array}$$

where $\hat{z}\left(t\right)$ is to account for the additive white Gaussian noise (AWGN). Since $f_{c}$ is usually very high compared to the bandwidth of the signal, in particular in the millimeter-wave band, the Doppler shift $\left(n\Delta f2v\right)/c_{0}$ is negligible for the subcarriers and the overall Doppler shift appears only caused by $\left(2vf_{c}\right)/c_{0}$.

The received signal is down-converted to baseband, which is equivalent to

(9) $$\begin{array}{cc} a(t) & =\sum_{\mu =0}^{M-1}\sum_{n=0}^{N-1}bS\left(\mu N+n\right)e^{\left(j2\pi n\Delta f(t-\frac{\left(2R\right)}{c_{0}}-\mu T_{s}-T_{\mathrm{cp}})\right)}e^{\left(j2\pi \frac{2vf_{c}}{c_{0}}t\right)}\mathrm{rect}\left(\frac{t-\left(\frac{2R}{c_{0}}\right)-\mu T_{s}}{T_{s}}\right)+z\left(t\right), \end{array}$$

where *b* and z(t) represent $\hat{b}\exp\left(-j2\pi f_{c}2R/c_{0}\right)$ and $\hat{z}\left(t\right)\exp\left(-j2\pi f_{c}t\right)$, respectively.

Finally, the signal a(t) is sampled, the CP part is removed before it is converted into the frequency domain by using FFT operation,

(10) $$\begin{array}{cc} A(\mu N+n) & =bS\left(\mu N+n\right)e^{\left(-j2\pi n\Delta f\frac{2R}{c_{0}}\right)}e^{\left(j2\pi \frac{2vf_{c}}{c_{0}}\mu T_{s}\right)}+Z\left(\mu N+n\right),\\ & \;n\in [0,N-1],\;\mu \in [0,M-1] \end{array}$$

where A(μN+n) and Z(μN+n) are frequency domain equivalents of a(t) and z(t).

Once the received signal is translated back into the frequency domain, the element-wise division of the received OFDM symbol by the respective transmitted OFDM symbol is performed to construct the channel matrix H, i.e.,

(11) $$H\left(\mu N+n\right)=be^{\left(-j2\pi n\Delta f\frac{2R}{c_{0}}\right)}e^{\left(j2\pi \frac{2vf_{c}}{c_{0}}\mu T_{s}\right)}+\frac{Z(\mu N+n)}{S(\mu N+n)},$$

where H(μN+n) represents the μth column and *n*th row of the channel matrix H, and Z(μN+n)/S(μN+n) defines the noise floor that depends on the digital modulation, e.g., a 16-QAM mapping affects the noise floor by approx 2.7 dB [36]. The IFFT of H, along subcarriers, provides the range information,

(12) $$\begin{array}{cc} r(d) & =e^{\left(j2\pi \frac{2vf_{c}}{c_{0}}\mu T_{s}\right)}\frac{b}{N}\sum_{n=0}^{N-1}e^{\left(-j2\pi n\Delta f\frac{2R}{c_{0}}\right)}e^{\left(j\frac{2\pi }{N}nd\right)}+\overset{ˇ}{z}\left(d\right),\\ & \;\;d\in [0,N-1] \end{array}$$

where $\overset{ˇ}{z}\left(d\right)$ represents the noise part.

The |r(d)| shows a peak value under the following condition:

(13) $$d=\left\lfloor \frac{2R\Delta fN}{c_{0}}\right\rfloor ,$$

i.e., the value of *d* corresponding to the maximum of |r(d)| holds the information of the target range, and the range resolution ΔR (minimum distinguishable distance between the two targets) is defined as

(14) $$\Delta R=\frac{c_{0}}{2\Delta fN}.$$

Similarly, the FFT operation over different OFDM symbols in H (over μ domain in (11)) provides the information about the speed of the target and can be recognized by using

(15) $$p=\left\lfloor \frac{2vf_{c}T_{s}M}{c_{0}}\right\rfloor ,\;p\in \left[0,M-1\right]$$

whereas the speed resolution Δv can be calculated by setting p=1,

(16) $$\Delta v=\frac{c_{0}}{2f_{c}T_{s}M}.$$

The IFFT/FFT operations on H provide processing gain due to coherent addition of signals, and the overall processing gain is

(17) G=NM.

If there are Ń guardband subcarriers on each side of the OFDM symbol, then the processing gain reduces to (N−2Ń)M. Here, it is important to note that the guardband subcarriers reduce the bandwidth of the OFDM waveform, and consequently the value of ΔR increases in (14) when *N* is replaced by (N−2Ń). Although ΔR increases due to guardband subcarriers, improvement in the range accuracy (resolution of IFFT) is linked to the size of IFFT [37].

**Figure 1:**
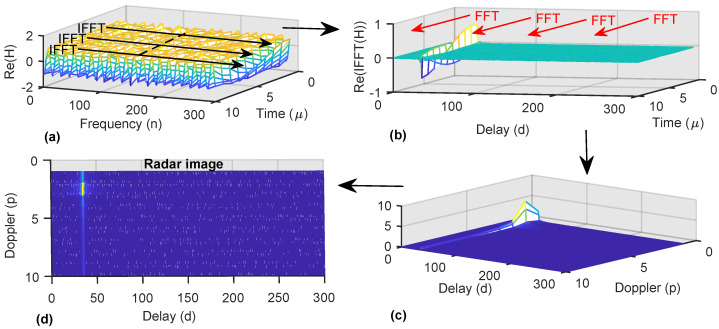
OFDM–based sensing from the channel matrix H. (**a**) IFFT operation over subcarriers. (**b**) FFT operation over OFDM symbols. (**c**) 3–D plot of the delay–Doppler profile. (**d**) Radar image, indicating the delay associated to the range and the Doppler frequency related to the speed of the target.

Figure 1 highlights the sensing processing using H. Figure 1a shows the real part of the channel matrix H, which has sinusoidal variations due to the range and speed of a single target. The IFFT operation, along with subcarriers, identifies the delay associated with the range, as shown in Figure 1b. Afterwards, the FFT operation provides the sensing information in the delay-Doppler profile, as shown in Figure 1c,d.

It is clear that the sensing performance depends on the OFDM waveform parameters because bandwidth defines the range resolution, and the duration of the OFDM waveform determines speed resolution. For the speed, the upper limit is selected as $\Delta f>20f_{c}v/c_{0}$ to maintain the acceptable level of orthogonality among the subcarriers [35].

## 3. Proposed Method for Range Extension

In OFDM-based sensing, the maximum range is limited by the CPI [28,35] as echoes falling outside the CPI cause ISI and suffer in processing gain. We propose a zero-delay shift method to compensate for delay τ in an echo to maintain its processing gain *G* during IFFT operation for OFDM-based sensing.

Using sampling intervals $\Delta T=1/F_{s}$ and Δf=1/(NΔT), the sampled version of (9) is

(18) $$\begin{array}{cc} a(k\Delta T) & =\sum_{\mu =0}^{M-1}\sum_{n=0}^{N-1}bS\left(\mu N+n\right)e^{\left(j2\pi n\frac{1}{\left(N\Delta T\right)}\left(k\Delta T-\frac{2R}{c_{0}\Delta T}-\mu \frac{T_{s}}{\Delta T}-\frac{T_{\mathrm{cp}}}{\Delta T}\right)\right)}\\ & \;\cdot e^{\left(j2\pi \frac{2vf_{c}}{c_{0}}k\Delta T\right)}\mathrm{rect}\left(\frac{k\Delta T-\frac{2R}{c_{0}}-\mu \frac{T_{s}}{\Delta T}}{\frac{T_{s}}{\Delta T}}\right)+z\left(k\Delta T\right), \end{array}$$

where *k* is the sampled time index. Using $F_{s}/\Delta f=T/\Delta T=N$, $T_{s}/\Delta T=N_{s}$, $T_{\mathrm{cp}}/\Delta T=N_{\mathrm{cp}}$, $m=\left\lfloor \left(2R\right)/\left(c_{0}\Delta T\right)\right\rfloor$, and a(k), z(k) to represent a(kΔT) and z(kΔT) respectively,

(19) $$\begin{array}{cc} a(k) & =\sum_{\mu =0}^{M-1}\sum_{n=0}^{N-1}bS\left(\mu N+n\right)e^{\left(j2\pi \frac{n}{N}\left(k-m-\mu N_{s}-N_{\mathrm{cp}}\right)\right)}\\ & \;\cdot e^{\left(j2\pi \frac{2vf_{c}}{c}k\Delta T\right)}\mathrm{rect}\left(\frac{k-m-\mu N_{s}}{N_{s}}\right)+z\left(k\right).\\ & \;k\in [0,MN_{s}-1] \end{array}$$

A delay-shift in *k* by *m* samples shifts the target at zero on the delay axis. Since *m* is unknown, sequentially increasing the delay-shift in *k* identifies *m* when a peak appears at delay zero. This process can identify echoes with delay longer than the CP, provided they arrive with detectable signal strength. If we extend the sensing range up to *Q* number of OFDM symbols, the proposed method can be described in following steps:

(1) *N* samples are selected (window of length *N*) from the received OFDM waveform to perform an *N*-point FFT operation;
(2) Received data symbols are divided by the transmitted data symbols in the current and previous Q−1 OFDM symbols;
(3) *N*-point IFFT operation is performed on results obtained in step 2, which provides first columns (in the delay domain) for *Q* number of matrices;
(4) Steps 1–3 are repeated for $N_{s}$ number of delay-shifts in the selected window. Completion of this step provides *Q* number of matrices each of size $N\times N_{s}$ in delay and delay-shift domains;
(5) Delay-zero rows (d=0) of each of the *Q* number of matrices, generated in step 4, are concatenated to form a row of another matrix, which will be used for range/speed processing;
(6) At this point, the selected window of *N* samples has been shifted by $N_{s}$ samples; now, OFDM symbols, which are used for division in step 2, are replaced by next OFDM symbol, e.g., $\left[S_{q},S_{q-1},\cdots ,S_{q-(Q-1)}\right]$ are replaced by $\left[S_{q+1},S_{q},\cdots ,S_{q-(Q-2)}\right]$, where $S_{q}$ denotes *q*th OFDM symbol;
(7) Steps 1–6 are repeated *M* times.

Using the above process, a matrix (in the delay-shift and time domains) of size $QN_{s}\times M$ is constructed, which requires an *M*-point FFT operation to complete the range/speed plot.

**Figure 2:**
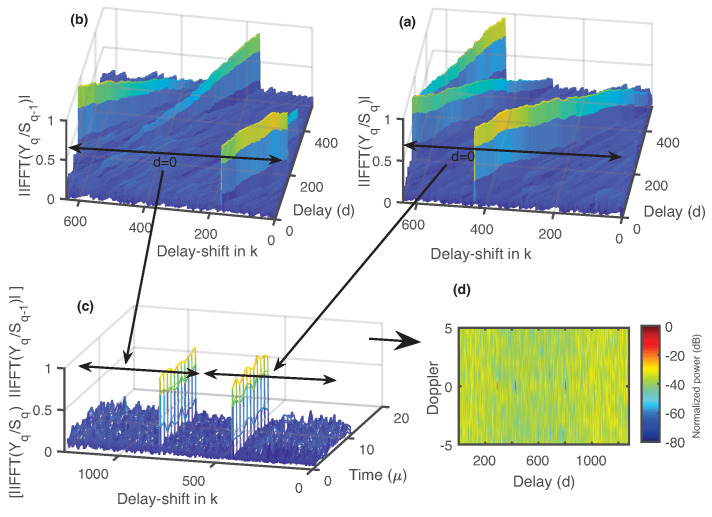
Explanation of the proposed method when range extension is up to two OFDM symbols. (**a**,**b**) Steps 1–4 of the proposed method provide two matrices. (**c**) Delay–zero rows of the matrices in (**a**,**b**) are concatenated according to the step 5. (**d**) Range/speed plot is completed using *M*–point FFT operation over time domain.

**Figure 3:**
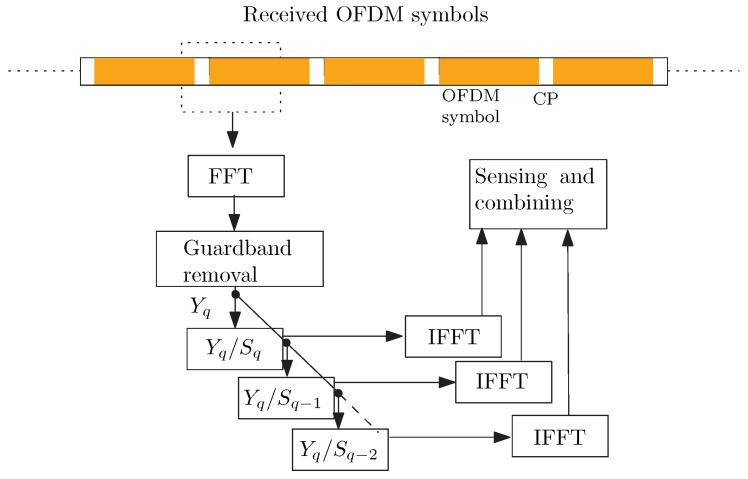
Schematic of the proposed range extension method to detect targets beyond the range limit in the conventional OFDM–based sensing.

Figure 2 shows different steps in the proposed method to detect two targets separated by more than one OFDM symbol duration; Q=2 is used, and the current OFDM symbol number is *q*. Figure 2a,b are obtained using steps 1–4 of the proposed method; Figure 2c is the plot of step 5 and using M=12 for step 6; Figure 2d is the final range/speed plot, where the range is extended up to two OFDM symbols. A schematic diagram of the proposed method is presented in Figure 3. Delay shifts are used in the sampled version of the incoming OFDM waveform to get the frequency domain signal $Y_{q}$. Element-wise division of $Y_{q}$ is performed with the current OFDM symbol $S_{q}$ and previous OFDM symbols for sensing and combining.

We use periodogram to compare the performance of the proposed method with conventional OFDM-based sensing. Periodogram of the conventional OFDM-based sensing is defined as [31],

(20) $$\begin{array}{cc} D_{\mathrm{conv}(d,p)}= & {\left|\sum_{\mu =0}^{M-1}r(d)e^{\frac{-j2\pi \mu p}{M}}\right|}^{2},\\ & \;d\in [0,N-1]\;p\in [0,M-1] \end{array}$$

where r(d) is defined in (12). A target is detected if the peak in $D_{\mathrm{conv}(d,p)}$ is above a threshold level (usually defined by the minimum detectable signal strength). For the range/speed plot, D is often transformed to normalized power, and in dB scale using $10\log_{10}\left(D/max\left[D\right]\right)$, where $max\left[D\right]$ represents the maximum value of D.

Similarly, the periodogram of the proposed method is

(21) $$D_{\mathrm{pro}(\hat{d},p)}={\left|\sum_{\mu =0}^{M-1}{\hat{r}}_{\hat{d}}\left(0,\mu \right)e^{\frac{-j2\pi \mu p}{M}}\right|}^{2},\;\hat{d}\in \left[0,\left(Q-1\right)N_{s}-1\right]\;p\in \left[0,M-1\right]$$

where $\hat{d}$ represents the delay-shift domain and ${\hat{r}}_{\hat{d}}\left(0,\mu \right)$ is obtained by concatenation of *Q* segments as defined in step 5 of the proposed method, i.e.,

(22) $$\begin{array}{cc} {\hat{r}}_{\hat{d}}\left(0,\mu \right) & =\left[r_{\overset{´}{d}}\left(0,\mu \right),r_{\overset{´}{d}}\left(0,\mu -1\right),...,r_{\overset{´}{d}}\left(0,\mu -\left(Q-1\right)\right)\right],\;\overset{´}{d}\in \left[0,N_{s}-1\right] \end{array}$$

where $r_{\overset{´}{d}}\left(0,\mu \right)$ is obtained by using d=0 in $r_{\overset{´}{d}}\left(d,\mu \right)$,

(23) $$\begin{array}{cc} r_{\overset{´}{d}}\left(d,\mu \right) & =\frac{1}{N}\sum_{n=0}^{N-1}H_{\overset{´}{d}}\left(\mu N+n\right)e^{\frac{j2\pi nd}{N}},\;d\in \left[0,N_{s}-1\right] \end{array}$$

i.e.,

(24) $$r_{\overset{´}{d}}\left(0,\mu \right)=\frac{1}{N}\sum_{n=0}^{N-1}H_{\overset{´}{d}}\left(\mu N+n\right).$$

If we represent $m=gN_{s}+\hat{m}$ where g∈[0,Q−2] and $\hat{m}\in \left[0,N_{\mathrm{cp}}-1\right]$, received *q*th OFDM symbol and transmitted OFDM symbols provide

(25) $$H_{\overset{´}{d},\left(q-i\right)}=\frac{Y_{\overset{´}{d},q}}{S_{\left(q-i\right)}},$$

where $H_{\overset{´}{d},\left(q-i\right)}$ is taken as simplified notation for $H_{\overset{´}{d}}\left(\left(q-i\right)N+n\right)$, i∈[0,Q−1], and $Y_{\overset{´}{d},q}=\mathrm{FFT}\left(A_{\overset{´}{d}}\left(k\right)\right)$, where $A_{\overset{´}{d}}\left(k\right)$ is the delay-shift of A(k) by $\overset{´}{d}$ and $k\in [\overset{´}{d}+\left(q\right)N_{s}-gN_{s}-\hat{m}+,\overset{´}{d}+\left(q+1\right)N_{s}-gN_{s}-\hat{m}-N_{\mathrm{cp}}]$ (interval of the N-samples of the waveform is selected at the initial step of the proposed method). It is clear that at $\overset{´}{d}={\overset{´}{d}}_{0}=\hat{m}+N_{\mathrm{cp}}$, $A_{\overset{´}{d_{0}}}\left(k\right)$ represents (q−g)th OFDM symbol without $N_{\mathrm{cp}}$; hence, (25) changes to

(26) $$H_{\overset{´}{d_{0}},\left(q-i\right)}=\frac{bS_{\left(q-g\right)}+Z_{\left(q-g\right)}}{S_{\left(q-i\right)}},$$

where $Z_{\left(q-g\right)}$ represents noise part in the (q−g)th OFDM symbol. Similarly, at $\overset{´}{d}={\overset{´}{d}}_{1}=\left(\hat{m}+N\right)$, $A_{\overset{´}{d}}\left(k\right)$ consists of last $N_{\mathrm{cp}}$ samples of the (q−g)th OFDM symbol and $N-N_{\mathrm{cp}}$ samples of (q−g+1)th OFDM symbol; therefore,

(27) $$H_{{\overset{´}{d}}_{1},\left(q-i\right)}=\frac{N-N_{\mathrm{cp}}}{NS_{\left(q-i\right)}}bS_{\left(q-g\right)}e^{\frac{-j2\pi n}{N}N_{\mathrm{cp}}}+\frac{N_{\mathrm{cp}}}{NS_{\left(q-i\right)}}bS_{\left(q-g+1\right)}+\frac{Z_{\left(q-g\right)}}{S_{\left(q-i\right)}}.$$

Using (26) in (24) provides the maximum of $r_{\overset{´}{d}}\left(0,\mu \right)$, which is same as r(d) in (20), whereas (27) indicates the additional peak with height reduced by a factor of $N_{\mathrm{cp}}/N$ and affected by the ISI. Similar to $D_{\mathrm{conv}(d,p)}$, where a processing of NM is assigned to a peak, $D_{\mathrm{pro}(\hat{d},p)}$ also provides the same processing gain when i=g in (26); otherwise, $H_{\overset{´}{d},\left(q-i\right)}$ in (26) is interference. In (27), contrary to interference term, $N_{\mathrm{cp}}$ samples are coherently added when used in (24) and the processing gain is $10\log_{10}\left(N_{\mathrm{cp}}^{2}/\left(N-N_{\mathrm{cp}}\right)\right)$.

In a generalized scenario, there can be *L* echoes with delays not limited to CPI; the received signal y(k) is the summation of all echoes, each represented by the (19),

(28) $$\begin{array}{cc} y(k) & =\sum_{l=0}^{L-1}\sum_{\mu =0}^{M-1}\sum_{n=0}^{N-1}b_{l}S\left(\mu N+n\right)e^{\left(j2\pi \frac{n}{N}\left(k-m_{l}-\mu N_{s}-N_{\mathrm{cp}}\right)\right)}\\ & \;\cdot e^{\left(j2\pi \frac{2v_{l}f_{c}}{c}k\Delta T\right)}\mathrm{rect}\left(\frac{k-m_{l}-\mu N_{s}}{N_{s}}\right)+z\left(k\right), \end{array}$$

where $m_{l}$ represents the delay associated with *l*th echo. Based on the delay $m_{l}$, we split the y(k) into three portions such as $y_{1}\left(k\right)$ for $m_{l}\leq N_{\mathrm{cp}}$, $y_{2}\left(k\right)$ for $N_{\mathrm{cp}}<m_{l}\leq N_{s}$, and $y_{3}\left(k\right)$ for $m_{l}>N_{s}$, i.e.,

(29) $$y\left(k\right)=y_{1}\left(k\right)+y_{2}\left(k\right)+y_{3}\left(k\right)+z\left(k\right).$$

Since $y_{3}$ is formed by the summation of echoes that are outside the current OFDM symbol, therefore this part is only ISI. Unlike $y_{3}\left(k\right)$, the ISI part of $y_{2}\left(k\right)$ increases as $m_{l}$ approaches to $N_{s}$. The detection of the echoes in $y_{2}\left(k\right)$ and $y_{3}\left(k\right)$ is possible if the processing gain *G* is sufficient to overcome the related ISI and noise.

### 3.1. Signal-to-Interference Ratio

In the proposed method, the signal-to-interference ratio (SIR) changes with the shifting of *k*. At the sensing receiver, strength of an echo depends on several factors such as antenna gain, round trip distance from the target, carrier frequency, and radar cross-section, as mentioned in (6). If we define $P_{{\mathrm{Rx}}_{1}}$ as the received power of $y_{1}\left(k\right)$, $P_{{\mathrm{Rx}}_{2}}$ for $y_{2}\left(k\right)$, and $P_{{\mathrm{Rx}}_{3}}$ for $y_{3}\left(k\right)$, then the SIR during the shifting of *k* is as below.

- At the beginning, echoes within $y_{1}\left(k\right)$ are detected under the collective ISI caused by $y_{3}\left(k\right)$ and $y_{2}\left(k\right)$, and we can define the SIR during this process as
  (30) $${\mathrm{SIR}}_{1}=\frac{P_{{\mathrm{Rx}}_{1}}}{\alpha_{2}\left(ḱ\right)P_{{\mathrm{Rx}}_{2}}+P_{{\mathrm{Rx}}_{3}}},$$
  where ḱ indicates the shift in *k* and $0\leq \alpha_{2}\left(ḱ\right)<1$ defines the part of $P_{{\mathrm{Rx}}_{2}}$ appearing as interference. $\alpha_{2}\left(ḱ\right)$ = 0 indicates that there are no echoes to form $y_{2}\left(k\right)$.
- At the second stage, when ḱ is beyond the $N_{\mathrm{cp}}$ and within $N_{s}$, echoes that form $y_{2}\left(k\right)$ are detected and a part of $y_{1}\left(k\right)$ causes ISI, which increases with ḱ. The SIR can be defined as
  (31) $${\mathrm{SIR}}_{2}=\frac{P_{{\mathrm{Rx}}_{2}}}{\alpha_{1}\left(ḱ\right)P_{{\mathrm{Rx}}_{1}}+P_{{\mathrm{Rx}}_{3}}},$$
  where $0\leq \alpha_{1}\left(ḱ\right)<1$ is used to account for the ISI caused by part of $P_{{\mathrm{Rx}}_{1}}$.
- Similarly, when we detect echoes in $y_{3}\left(k\right)$, the SIR is
  (32) $${\mathrm{SIR}}_{3}=\frac{P_{{\mathrm{Rx}}_{3}}}{P_{{\mathrm{Rx}}_{1}}+\alpha_{2}\left(ḱ\right)P_{{\mathrm{Rx}}_{2}}},$$
  where $P_{{\mathrm{Rx}}_{1}}$ appears as ISI because at this stage the element-wise division is performed by the previous OFDM symbol $S_{q-1}$.

Here, it is important to mention that $P_{{\mathrm{Rx}}_{1}}>P_{{\mathrm{Rx}}_{2}}>P_{{\mathrm{Rx}}_{3}}$ (assuming same radar cross-section for different targets associated with echoes) because of the FSPL difference between echoes that form $y_{1}\left(k\right)$, $y_{2}\left(k\right)$ and $y_{3}\left(k\right)$. Therefore, ${\mathrm{SIR}}_{1}>{\mathrm{SIR}}_{2}>{\mathrm{SIR}}_{3}$, which clearly indicates that detection of echoes in $y_{2}\left(k\right)$ and $y_{3}\left(k\right)$ is not possible without the sufficient processing gain obtained through the IFFT/FFT operation during sensing. Usually, the Doppler estimation requires large interval (compared to the OFDM symbol duration) of the waveform; therefore, large number of OFDM symbols can provide sufficient processing gain for the echoes to overcome ISI.

### 3.2. Effect of CP

For $y_{3}\left(k\right)$, during delay-shifting stage of the proposed method, the last part of the window, which is selected in step 1 of the proposed method, occupies the complete CP part of the OFDM symbol, and a peak with processing gain of $20\log_{10}\left(N_{\mathrm{cp}}\right)-10\log_{10}\left(N-N_{\mathrm{cp}}\right)-{\mathrm{Mod}}_{\mathrm{noise}}$ dB appears at delay zero (defined by (27)). Where ${\mathrm{Mod}}_{\mathrm{noise}}$ dB is the raise in noise due to digital modulation, e.g., 16-QAM causes a raise of ≈2.7 [36]. For *M* number of OFDM symbols, an additional $10\log_{10}\left(M\right)$ dB is added to CP peak. Appearance of peaks due to CP, for echoes with delay longer than OFDM symbol duration, which are exactly *N* samples behind the target, can be eliminated from the observations.

### 3.3. Computational Complexity of the Proposed Method

The computational complexity is measured in terms of number of complex multiplications and additions. It is considered that removal of the CPI is negligible in complexity, divisions are equivalent to multiplications, and performing an IFFT/FFT of size *N* requires $\left(N/2\right)\log_{2}\left(N\right)$ number of complex multiplications and $N\log_{2}\left(N\right)$ number of complex additions [27]. Table 1 provides the complexity of the proposed method and the conventional OFDM-based sensing. The complexity of the proposed method is higher by a factor of ≈$QN_{s}$ because whole chain of operation, for range detection, is performed after each shift in *k* with maximum shifts as $QN_{s}$. For speed, *N* number of *M*-point FFTs is increased to $QN_{s}$.

## 4. Simulation Results

To verify the processing of the proposed OFDM-based converged system, a baseband equivalent model is implemented and simulated in MATLAB. An impulse response, having taps at the round-trip delay of the targets, and each tap varying over OFDM symbols according to the complex exponential of the Doppler frequency, is used to represent the sensing channel, whereas SNR =15 dB is set for simulation. Equal signal strength is used for different targets, while other parameters, listed in Table 2, are selected to match parameters used in our experiment.

**Figure 4:**
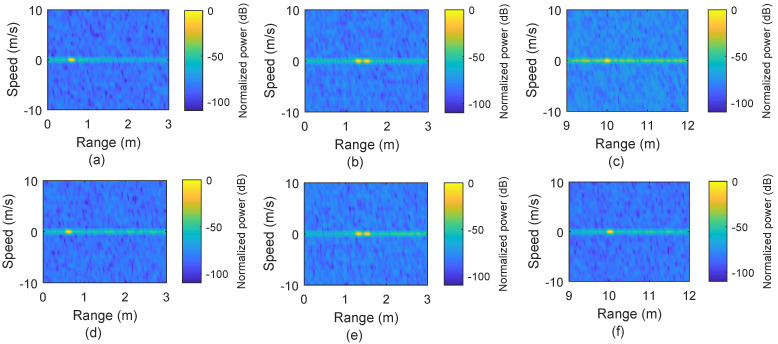
Simulation results of the conventional OFDM–based sensing vs. the proposed range extension method. (**a**–**c**) Range plots for a target at 0.6 m, two targets at 1.3 m and 1.5 m, and a target at 10 m, respectively, by using conventional OFDM–based sensing. (**d**–**f**) Range plots obtained by using the proposed range extension method.

It is important to note that for scenarios where we use only static targets, M=36 is used, which provides sufficient gain for range detection but results in a high value of Δv in the range/speed plot, although this is irrelevant for static targets. For the range/speed plot, the absolute of the delay/Doppler matrix is first normalized to unit (by dividing with the maximum absolute value) and then converted to dB scale. Figure 4a–c presents the range plots for static targets at distances 0.6 m, 1.3 m 1.5 m, and 10 m, respectively, using the conventional OFDM-based sensing. Results show that the targets are identified correctly at the distances in Figure 4a,b.

However, Figure 4c clearly shows that the sensing performance has been compromised, in terms of SNR, for the target located at a 10 m distance. This reduction of SNR happens because the CPI covers a range up to 3.2 m. Beyond this range limit, ISI occurs, and the processing gain is also reduced for that target located at 10 m. In comparison, the scenario mentioned above is also processed for sensing using our proposed method by finding the zero-delay for each target, as shown in Figure 4d–f. Our proposed method offers better performance for the target at 10 m by avoiding the loss in processing gain.

**Figure 5:**
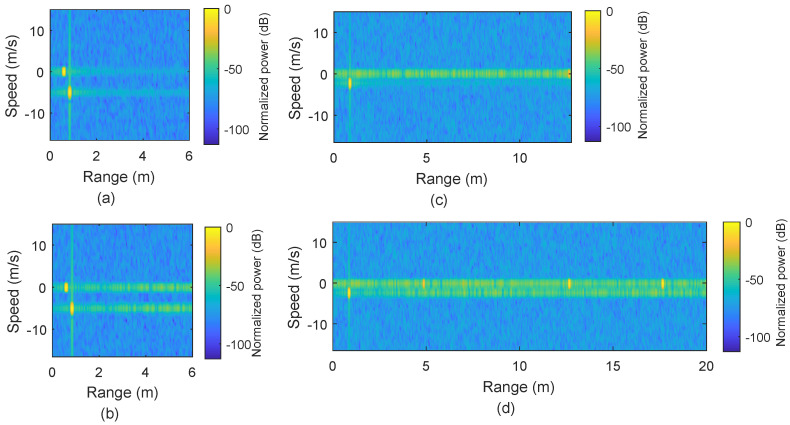
(**a**) One static target at 0.6 m and one moving with 5 m/s using the conventional OFDM–based sensing. (**b**) Results by using the proposed range extension method for the scenario in (**a**). (**c**) Range–speed plot for targets at 0.85 m and moving with 2.34 m/s, with second and third static targets at 12.65 m and 17.65 m, respectively. (**d**) Range–speed plots obtained by using the proposed range extension method for targets mentioned in (**c**).

Figure 5 represents the results when multiple targets exist and one of them is moving. In Figure 5a, the conventional approach provides accurate results for two targets, one static target located at 0.6 m and the other target moving with a speed of 5 m/s and situated at 0.85 m. In comparison to Figure 5a, Figure 5b represents the results obtained by using our proposed method where peak height is similar to the peak height in Figure 5a, but ISI effect is used for locations away from the targets.

In order to verify the proposed method for the range beyond the OFDM symbol, we also simulated the case that three targets are placed at 0.85 m (moving with 2.34 m/s), 12.65 m, and 17.65 m, respectively, and the sensing results are shown in Figure 5c,d. The farthest target at 17.65 m is beyond the range of an OFDM symbol duration (12.77 m) and it does not appear in Figure 5c using the conventional approach, but it is detectable with our proposed method, as shown in Figure 5d. There is another peak with height ≈−20 dB at 4.88 m (12.77 m behind the target at 17.65 m) in Figure 5d, which appears due to the CP effect.

## 5. Experimental Setup and Results

**Figure 6:**
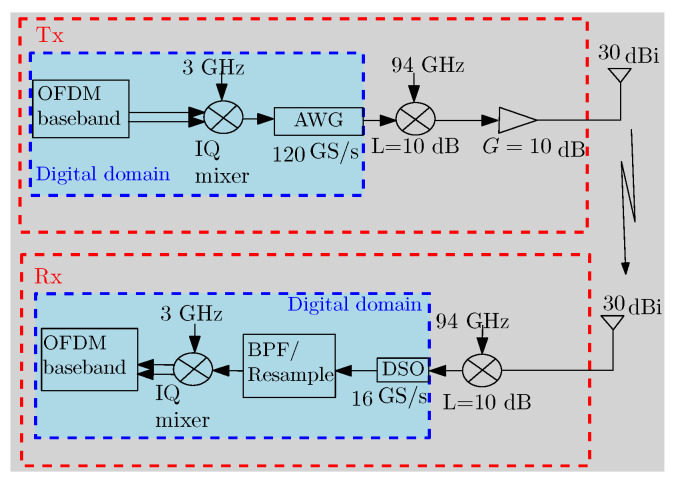
Block diagram of the measurement setup, showing different stages of processes in the digital and analog domains at the transmitter $T_{X}$ and the receiver $R_{X}$.

In this work, we also implement an experimental demonstration. Figure 6 shows the configuration of our system in the experiment, which is composed of several blocks in the digital and analog domain for transmission and reception. In our experiment, the baseband 16-QAM OFDM waveform is digitally generated for communication and sensing using parameters listed in Table 2. An oversampling factor of 20 is used before the signal is digitally up-converted to an intermediate frequency (IF) at 3 GHz through the IQ mixing. The IF signal is then fed to an arbitrary waveform generator (AWG) operating at 120 GS/s. Before the free-space transmission, the signal is up-converted to the W-band with a carrier frequency of 97 GHz using a commercially available W-band mixer at 94 GHz. Subsequently, a W-band amplifier with 10 dB gain is used to boost the signal to around 0 dBm, and a pair of conical horn antennas with a gain of 30 dBi is used for transmission and reception. At the receiver, the signal is first down-converted into the IF domain using a similar W-band mixer, sampled using a digital sampling oscilloscope (DSO) (KEYSIGHT MXR608A, sampling rate of 16 GS/s, bandwidth of 6 GHz) and then processed digitally to retrieve the baseband signal for further processing. In the digital domain, typical Fourier sidelobes are suppressed by using the Hamming window.

**Figure 7:**
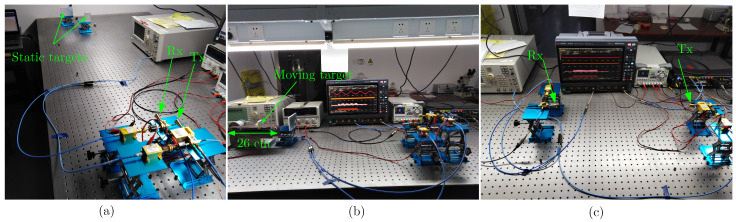
Experimental setup for the measurements. (**a**) Photo of the setup to measure range of two static targets. (**b**) Range-speed measurement setup of a static target and a target mounted on a belt capable to move with adjustable speed up to 5 m/s. (**c**) Data link arrangement.

The photos of the experimental setup are shown in Figure 7a–c. Figure 7a shows the arrangement for realizing the reflective sensing to measure the range of two static targets with flat reflective surfaces. Figure 7b shows the setup for speed and range measurement with a static target and a moving target. The speed is measured via a reflective target mounted on a belt, which moves the target along the LOS and away from the receiver with adjustable speed. In this case, the Doppler frequency shift is induced in the signal reflected from the moving target, and the down-conversion from W-band carrier frequency to an IF yields a sinusoidal of the Doppler frequency, which can be observed at the DSO. Observation of the sinusoidal-like variations in the received signals at the DSO indicates the correct measurement arrangement for speed measurement.

The setup is calibrated during range measurement, and we measure different cases for illustrating the range and speed measurements, as listed in Table 3, where $d_{1}$ and $d_{2}$ are the actual distances of targets from the receiving antenna and ${\hat{d}}_{1}$, ${\hat{d}}_{2}$ represent measurement results. Similarly, *v* and $\hat{v}$ in Table 3 represent actual and measured speeds, respectively. For the data transmission part, a LOS link is established by placing the receiver at the location of the static target, as shown in Figure 7c. In the following subsections, we discuss experimental results obtained from the measurements.

### 5.1. Range Measurement

**Figure 8:**
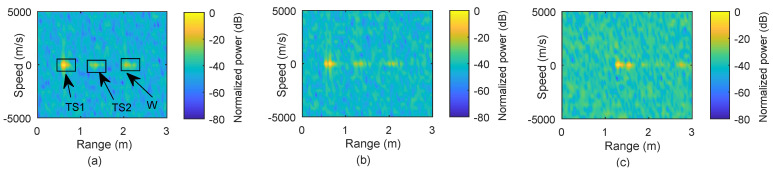
Measurement results for different arrangements of static targets. (**a**) Range plot of a target when placed at 0.6 m. (**b**) Range plot for two targets placed at 0.65 m and at 0.6 m. (**c**) Results of two targets placed at 1.313 m and at 1.548 m, respectively.

In Figure 8a, range measurement is implemented when a static target is placed 0.605 m from the receiver. The target is detected with an error of 0.005 m, as mentioned in Table 3. Based on Figure 8a, we can also observe multiple reflections originating from the target and wall; TS1 and TS2 indicate first and second reflections from the target, whereas W is the wall’s reflection. Here, the second reflection refers to a signal that is reflected twice from the target and eventually detected by the receiver since the receiver reflects a portion of strong signal. Then, we perform the measurements for two targets placed at 0.6 m and 0.65 m, and Figure 8b shows that the closely placed targets are distinguishable from each other at their locations. Similarly, Figure 8c provides the measurement results for two targets standing at 1.313 m and 1.548 m, respectively. The change in the received power is due to a higher FSPL.

### 5.2. Range Extension

**Figure 9:**
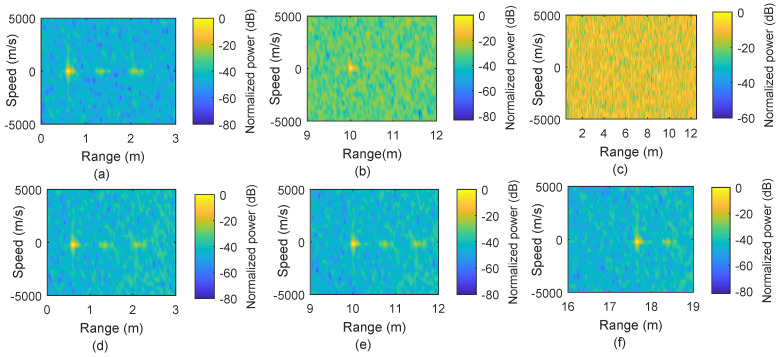
Range extension of the measurement results, where the range is extended through offset–delay. (**a**–**c**) Results of conventional approach for targets at 0.6 m, 10 m, and 17.65 m, respectively. (**d**–**f**) Results obtained through the proposed range extension method for the arrangements as for results in (**a**–**c**).

In this case, our proposed range extension method is applied using digital delay offset in the received signal to realize larger range values such as 10 m. In Figure 9, ranging performance using the conventional (Figure 9a–c) and the proposed method (Figure 9d–f) is shown, when the target location is within the CPI, within the OFDM symbol duration, and beyond the OFDM symbol duration.

Figure 9a is the range plot for the target placed at 0.6 m in the measurement, whereas Figure 9b shows the same target shifted to 10 m, which reduces processing gain and, in turn, performance degradation. Similarly, the target in Figure 9c is beyond the OFDM symbol duration and does not appear in the conventional OFDM-based processing. As a benefit, the results obtained through our proposed method are shown in Figure 9d–f, and all targets are identifiable for all the same cases. Multiple reflections from the target and reflection from the wall appear as mentioned in Figure 8a.

### 5.3. Speed Measurement

**Figure 10:**
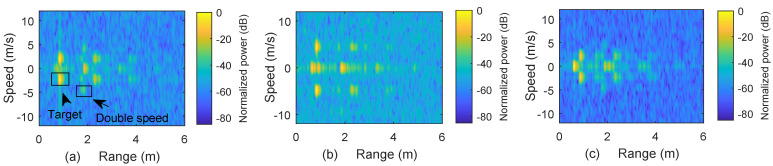
Range-speed plots for the speed measurements. (**a**) Range–speed plot of a single target moving with 2.34 m/s, whereas the moving assembler acts as a static unit. (**b**) Range–speed plot of the target in (**a**) with speed increased to 5 m/s. (**c**) Range–speed plot of a static target, placed at 0.6 m, and a moving target with speed of 2.34 m/s.

Figure 10 shows the measurement results for two different speeds. The speed of a single target (0.03×0.03 m^2^) is set to 2.34 m/s in Figure 10a, while Figure 10b shows the result for a single target (0.015×0.02 m^2^) moving at 5 m/s. The assembler, which contains the target mounted on a moving belt, is 0.26 m long and is placed 0.67 m from the receiving antenna. We can notice that both speed and distance can be identified, but due to the smaller size of the moving target, the received signal strength in Figure 10b is weaker than in Figure 10a.

The result in Figure 10c are obtained by simultaneously placing a static target at 0.6 m and a moving target (0.015×0.02 m^2^) at 2.34 m/s located between 0.67 m to 0.9 m. It is observed that the setup can accurately measure the speed in different arrangements within the speed resolution of 0.79 m/s and the range of the static target within the range resolution of 0.042 m. Measurements for the speed values in our experiment required an interval of 1.96 ms to capture sinusoidal variations caused by the Doppler frequency. Multiple reflections from the moving target cause echoes with higher speeds, as indicated in Figure 10a. The ± speed in the results is due to the use of a double-sideband W-band mixer for up/down conversion in the experiment. The ± speed ambiguity can be removed by using single sideband devices such as IQ modulators.

### 5.4. Data Communication

**Figure 11:**
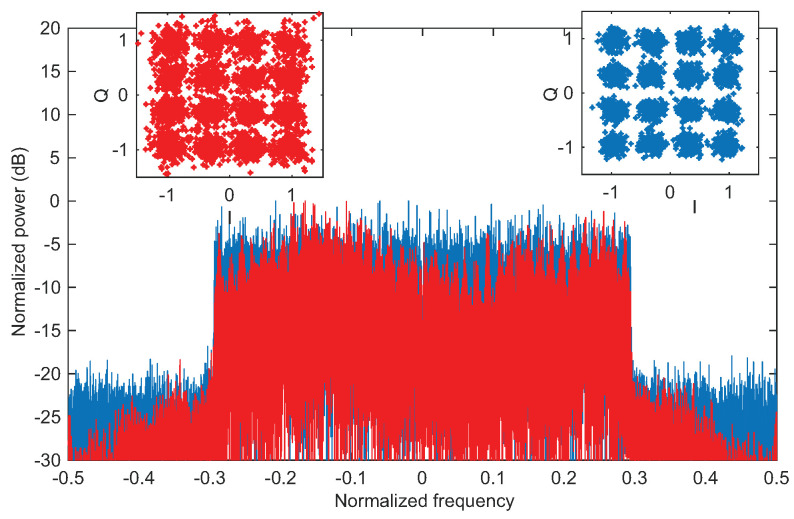
Data transmission results. The baseband spectrum and 16–QAM constellation, and red color for a LOS link of 0.6 m with background reflections. Blue color is the corresponding simulation result.

To demonstrate the convergence of communication and sensing by using the same 16-QAM OFDM waveforms, we also measured data communication performance in terms of BER. A subframe wise processing is used, where 12 OFDM symbols form a subframe, and each OFDM symbol has 300 active subcarriers. In one subframe, four OFDM symbols are multiplexed with pilot subcarriers (4×50 pilot subcarrier in a subframe), and a code rate of 0.76 is used. In the experiment, we placed the receiver and transmitter in the LOS link distance of 0.6 m, with a bit rate of 8.08 Gbps. Due to non-ideal environment for the measurement such as limited dimensions of the lab and surrounding objects, multiple reflections of the transmitted signal arrive at the receiving antenna. Changes in the spectrum of the received signal in Figure 11 (red color) confirm the presence of the multiple reflections. Therefore, channel equalization is necessary to recover the transmitted data, which increases BER compared to AWGN channel [38]. We performed frequency domain equalization (zero-forcing) by using the transmitted pilot subcarriers for the channel estimation. As a result, a BER of 0.01 is recorded by comparing the transmitted and received data bits, when the average received SNR is around 15 dB, which is below the soft-decision forward error correction (SD-FEC) threshold of $1.5\times 10^{-2}$ [39]. As a comparison, a BER of $3.6\times 10^{-4}$ is obtained in the simulation due to the absence of the background reflections. A comparison of the received spectrum and the corresponding constellations is provided in Figure 11, for illustration purposes.

### 5.5. Comparison with Existing Works

A performance comparison of the proposed system is compared with the existing OFDM-based works in Table 4. In most of the experimental demonstrations for the convergence of communications and sensing, the OFDM waveform is evaluated for the sensing performance only. Since the speed resolution is linked with the observation time, higher speed resolution can be realized with longer observation intervals, as shown in Table 4.

## 6. Conclusions

We demonstrated a W-band simultaneous communication and sensing system operating at 97 GHz using a common 16-QAM OFDM waveform. The zero-delay-shift-based approach is proposed to overcome the sensing range limitation in the conventional OFDM-based sensing systems and enable range extension for the OFDM-based converged system. Both simulation and experimental measurements are performed in the W-band with a bandwidth of 3.9 GHz. Due to the large bandwidth available in the W-band, we achieve a sensing resolution of 0.042 m in range and 0.79 m/s in speed in the experiment. The target range well beyond the CPI is detected by using our proposed method. Furthermore, we also measure the 16-QAM OFDM communication performance, and the BER below the SD-FEC is achieved. The successful demonstration of the convergence of communication and sensing using the same waveform is a significant step towards future wireless applications.

## Figures and Tables

**Table 1:** Computational complexity of the proposed method.

OFDM-Based Sensing			Proposed Method		
Operation	Complex Multiplications	Complex Additions	Operation for QN_s_ Delays	Complex Multiplications	Complex Additions
Division to get H	$4MN_{ac}$	0	Division to get H	$4MN_{ac}QN_{s}$	0
*N*-point IFFT	$M\left(N/2\right)\log_{2}\left(N\right)$	$MN\log_{2}\left(N\right)$	*N*-point IFFT	$\left(QN_{s}\right)M\left(N/2\right)\log_{2}\left(N\right)$	$\left(QN_{s}\right)MN\log_{2}\left(N\right)$
*M*-point FFT	$N\left(M/2\right)\log_{2}\left(M\right)$	$MN\log_{2}\left(M\right)$	*M*-point FFT	$\left(QN_{s}\right)\left(M/2\right)\log_{2}\left(M\right)$	$\left(QN_{s}\right)M\log_{2}\left(M\right)$
Total	$\left(MN/2\right)\log_{2}\left(MN\right)$	$MN\log_{2}\left(MN\right)$	Total	$\left(QN_{s}\right)\left(M/2\right)(N\log_{2}\left(N\right)$	$\left(QN_{s}\right)M(N\log_{2}\left(N\right)$
				$+\log_{2}\left(M\right))$	$+\log_{2}\left(M\right))$

**Table 2:** Simulation parameters.

Sampling frequency $F_{s}$	6 GHz
Carrier frequency $f_{c}$	97 GHz
FFT size *N*	512
Subcarrier spacing Δf	11.71875 MHz
No. of active subcarriers $N_{\mathrm{ac}}$	300
Bandwidth *B*	3.9 GHz
Bandwidth occupied by active subcarriers $N_{\mathrm{ac}}\Delta f$	3.51 GHz
Bandwidth utilization	90%
Digital mapping	16 QAM
Effective OFDM symbol duration $T_{s}$	0.1066 μs
Cyclic prefix duration (CP)	128 samples, 0.0213 μs
No. of OFDM symbols *M*	36 for range 18,432 for speed
Range resolution $\Delta R=c/(2\Delta fN_{\mathrm{ac}})$	0.042 m
Unambiguous range (N−1)c/(2ΔfN)	12.775 m
Maximum range (within CPI)	3.2 m
Speed resolution Δv	0.79 m/s
Unambiguous speed $\pm \frac{(M-1)\Delta v}{2}$	±7464 m/s

**Table 3:** Number of targets and their arrangements for different scenarios.

	Actual Values				Measured Values			
Scenario	$d_{1}$	$v_{1}$	$d_{2}$	$v_{2}$	${\hat{d}}_{1}$	${\hat{v}}_{1}$	${\hat{d}}_{2}$	${\hat{v}}_{2}$
	m	m/s	m	m/s	m	m/s	m	m/s
Single static target	0.605	0	N.A	N.A	0.60	0	N.A	N.A
Two static targets	0.60	0	0.65	0	0.575	0	0.65	0
Two static targets	1.313	0	1.548	0	1.30	0	1.55	0
Single moving target	N.A	2.34	N.A	N.A	0.85	2.43	N.A	N.A
Single moving target	N.A	5	N.A	N.A	0.85	4.87	N.A	N.A
One static target, one moving target	0.6	0	N.A	2.34	0.6	0	0.875	2.43

**Table 4:** Performance comparison of the proposed converged system with the existing works.

Reference	Carrier Frequency	Bandwidth	Range Resolution	Observation Time	Speed Resolution	Data Rate
[34]	24 GHz	93 MHz	1.6 m	3.168 ms	1.97 m/s	20 Mbit/s
[31]	2.4 GHz	98.28 MHz	1.5 m	20 ms	4.2 m/s	Not evaluated
[32]	28 GHz	400 MHz	0.4 m	0.25 ms	N.A	Not evaluated
[28]	77 GHz	200 MHz	0.75 m	8 ms	0.48 m/s	Not evaluated
[29]	77 GHz	1.024 GHz	0.14 m	4.4 ms	0.38 m/s	Not evaluated
[30]	5.2 GHz	80 MHz	1.87 m	128 ms	0.22 m/s	Not evaluated
Proposed	97 GHz	3.9 GHz	0.04 m	1.9 ms	0.97 m/s	8.08 Gbit/s
